# An Exploration of Nurse Manager Leadership Styles and the Effect on Work Engagement Among Staff Nurses: A Mixed‐Method Study

**DOI:** 10.1002/nop2.70407

**Published:** 2026-02-21

**Authors:** Amal Alluhaybi, Kim Usher, Joanne Durkin, Amanda Wilson

**Affiliations:** ^1^ Faculty of Nursing Umm Al Qura University Makkah Saudi Arabia; ^2^ School of Nursing and Midwifery, Faculty of Health the University of Technology Sydney Sydney NSW Australia; ^3^ School of Health University of New England Armidale NSW Australia; ^4^ School of Nursing and Midwifery University of Newcastle Newcastle NSW Australia

## Abstract

**Aim:**

To provide a comprehensive understanding of how nurse managers' leadership styles affect staff engagement and to identify key themes that influence engagement from both statistical and experiential perspectives within a multicultural healthcare context.

**Design:**

Explanatory sequential mixed‐methods design, with integration occurring across the design, methods and reporting stages using the Pillar Integration Process.

**Methods:**

The study was conducted in four public hospitals in western Saudi Arabia. Quantitative data were collected from 278 registered nurses using the validated Multifactor Leadership Questionnaire (MLQ‐5X) and Utrecht Work Engagement Scale (UWES‐9). Thirteen nurses participated in follow‐up semi‐structured interviews. The study adhered to the Good Reporting of a Mixed Methods Study (GRAMMS) guidelines.

**Results:**

Four integrated themes emerged: relational leadership, recognition and reward, impact of neglectful leadership and cultural competence in leadership. Saudi and non‐Saudi nurses perceived leadership differently, influenced by cultural norms.

**Patient or Public Contribution:**

No patient or public contribution.

## 1 Introduction

Nurses constitute approximately 50% of the global healthcare workforce, yet the world faces a shortage of nearly four million nurses (WHO 2024). Declining recruitment and high turnover—often linked to burnout, job dissatisfaction and inadequate leadership support—exacerbate this issue (Buchan and Catton 2023). Research indicates that nursing shortages negatively impact both staff well‐being and patient outcomes (Dickson et al. 2024). One proposed solution to this shortage is enhancing the work engagement of existing nursing staff (Cai et al. 2023), as engaged nurses are more likely to remain in their roles and contribute positively to patient care (Cao et al. 2020; Li et al. 2019).

Work engagement, defined as a positive and fulfilling state of mind characterised by vigour, dedication and absorption, is essential for nurse retention and performance (Schaufeli et al. 2002; Schaufeli and Bakker 2004). Engaged nurses demonstrate higher organisational commitment, lower turnover and stronger contributions to patient outcomes (Cao et al. 2020; Li et al. 2019; Wei et al. 2023). In nursing, work engagement has gained attention as a strategy to address key healthcare challenges, including rising costs, increasing errors, global workforce shortages and growing expectations for care quality (Al Mamari and Groves 2023; Keyko et al. 2016). However, stress, burnout, absenteeism and low satisfaction continue to undermine nurse engagement (Van Bogaert et al. 2017).

Addressing these challenges requires effective leadership, as leadership influences workplace culture, job satisfaction and ultimately, nurse engagement (Bailey and Cardin 2018). The World Health Organization (WHO) estimates that an additional nine million nurses and midwives will be required by 2030 to meet global health demands (WHO 2020). Nurses, as frontline caregivers, play a critical role in influencing patient outcomes and overall healthcare quality (Boamah et al. 2018). Effective leadership is essential in fostering this engagement, as leadership not only provides direction but also inspires collaboration and improvement within healthcare teams (Abdullatif Ibrahim et al. 2023). The WHO recommends enhancing the leadership skills of nurse managers as a strategy to boost nursing engagement (WHO 2020).

Leadership, the ability to influence others to achieve common goals (Northouse 2021), is widely recognised as a key factor in enhancing nurse work engagement, improving job satisfaction and ultimately retaining a skilled workforce (Sfantou et al. 2017). Despite extensive research on leadership and nurse engagement, most studies rely on quantitative methodologies, which primarily measure statistical relationships but fail to capture nurses' lived experiences and the organisational and cultural nuances that shape engagement (Alluhaybi et al. 2023; Keyko et al. 2016). This gap is particularly evident in Saudi Arabia, where healthcare systems operate within a multicultural workforce (Alasiri and Mohammed 2022; Almutairi et al. 2015) influenced by both hierarchical organisational structures (Hofstede 2011) and sociocultural factors (Alsadaan et al. 2021). These intertwined elements create a unique environment in which leadership may be experienced and interpreted differently.

To address this gap, the present study explored how nurse manager leadership influences nurse engagement in Saudi Arabia. A sequential explanatory mixed‐methods design was employed, integrating statistical analysis with qualitative data to provide both empirical robustness and contextual depth. Quantitative analysis identifies statistical relationships, while qualitative insights explore nurses' perceptions and lived experiences (Tenny et al. 2025). This design addresses the limitations of single‐method studies by incorporating both numerical trends and nuanced, real‐world perspectives (Creswell and Clark 2018).

Building on earlier systematic reviews, quantitative studies (Alluhaybi et al. 2024, 2023) and a qualitative study (Alluhaybi et al. 2025), this paper presents an integrated analysis grounded in pragmatism. The aim is to provide a comprehensive understanding of how leadership styles—transformational, transactional and passive‐avoidant—affect nurse engagement within the Saudi context.

The primary research questions for this study were:

1. What is the relationship between leadership styles and work engagement among nurses in Saudi Arabia?
2. How do nurses perceive and experience different leadership styles in relation to their work engagement?
3. What leadership behaviours enhance or hinder nurse engagement within Saudi Arabia's unique cultural and organisational context?

By addressing these questions, this study aimed to provide valuable insights into effective leadership practices for managing a diverse nursing workforce in Saudi Arabia. The findings will help inform leadership training programmes, healthcare policies and organisational strategies to create a supportive and engaging work environment for nurses, ultimately enhancing patient care outcomes.

## 2 Method

### 2.1 Aims

This study explored the relationship between leadership styles and nurse engagement by integrating quantitative and qualitative findings. The specific objectives were to:

1. Examine the statistical relationship between transformational, transactional and passive‐avoidant leadership styles and nurse engagement.
2. Identify key themes from nurses' lived experiences regarding leadership styles in their workplaces.
3. Synthesise statistical trends and qualitative themes to develop evidence‐based recommendations for leadership practices that enhance nurse engagement

### 2.2 Design

This study employed an explanatory sequential mixed‐methods design. The quantitative phase assessed the relationship between leadership styles and nurse engagement, followed by a qualitative phase to explore participants' experiences. Integration occurred at the design, methodological and reporting levels using the Pillar Integration Process (Fetters et al. 2013). This study adhered to the GRAMMS (Good Reporting of a Mixed Methods Study) guidelines to ensure transparent and rigorous reporting of the mixed‐methods design (O'cathain et al. 2008).

#### 2.2.1 Integration Strategy

To ensure comprehensive synthesis, integration was carried out at three levels—design, methodology and reporting—based on the framework by Fetters et al. (2013).

**FIGURE 1:**
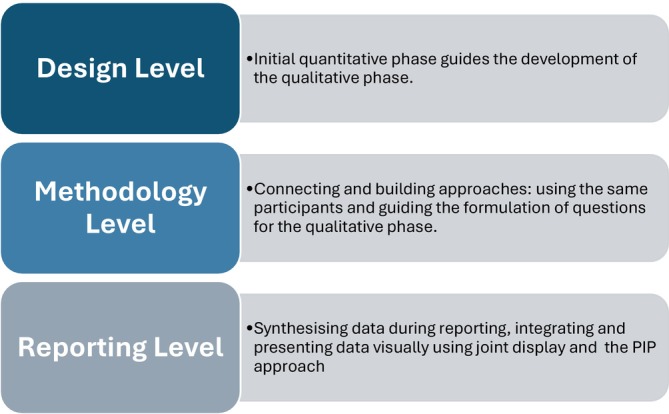
Levels of integration in mixed methods research.

This Figure 1 shows how integration occurs at three key levels in mixed methods research—Design, Methodology and Reporting—demonstrating how quantitative and qualitative phases are connected and synthesised throughout the study.

#### 2.2.2 Mixed Methods Integration at the Design Level

The explanatory sequential design allowed initial quantitative results to inform the qualitative phase. This ensured that qualitative data collection focused on areas requiring deeper insight.

#### 2.2.3 Mixed Methods Integration at the Methodology Level

Integration at this level was achieved through *connecting* (using the same sample or subgroup) and *building* (developing qualitative questions based on quantitative results), enhancing coherence between the two phases (Draucker et al. 2020; Fetters et al. 2013).

#### 2.2.4 Mixed Method Integration Reporting Level

Findings were merged using the Pillar Integration Process (PIP) and visually presented in a joint display table (Table 1). This facilitated the clear synthesis of statistical relationships and experiential data, supporting a richer interpretation of how leadership impacts nurse engagement in the Saudi context.

**TABLE 1:** Demographic characteristics of the participants.

Variable	Category	*n*	%
Gender	Male	51	18.3
	Female	227	81.7
Age group (years)	20–29	58	20.9
	30–39	176	63.3
	40–49	44	15.8
Nationality	Saudi	111	39.9
	Non‐Saudi	167	60.1
Years of experience	1–5 years	111	39.9
	6–10 years	87	31.3
	≥ 11 years	80	28.8
Department	Inpatient unit	216	77.7
	Outpatient unit	62	22.3

### 2.3 Sample

A sequential sampling approach was used to ensure alignment between the quantitative and qualitative phases. All participants were registered nurses with at least one year of experience and held non‐managerial positions.

- *Quantitative phase*: Convenience sampling was used to recruit 450 registered nurses from four referral hospitals, of whom 278 met the inclusion criteria and completed the survey (response rate: 61.8%).
- *Qualitative phase*: Purposive sampling was applied to select 13 nurses from the survey respondents who had indicated their willingness to participate in interviews. According to Creswell and Poth (2018), a sample of 10–15 participants is appropriate to achieve thematic saturation in relatively homogeneous qualitative samples. The selection ensured diverse representation based on department, nationality and years of experience, enhancing the depth of qualitative insights.

Given the multicultural workforce, the study ensured cultural appropriateness of the research instruments. The Multifactor Leadership Questionnaire (MLQ‐5X) and Utrecht Work Engagement Scale (UWES‐9) were administered in English, as it is the primary professional language in Saudi hospitals. No major modifications were made to these validated instruments, but their clarity and relevance were reviewed. In the qualitative phase, interviews were conducted in both English for non‐Arabic speakers and Arabic for Arabic speakers, allowing participants to express themselves comfortably, with translations cross‐verified to maintain accuracy and meaning.

### 2.4 Data Collection

#### 2.4.1 Quantitative Data Collection

This study employed a descriptive cross‐sectional correlational design to investigate the relationship between nurse managers' leadership styles and staff nurses' work engagement in four public hospitals in western Saudi Arabia. The study utilised two main instruments: the Multifactor Leadership Questionnaire (MLQ‐5X) and the Utrecht Work Engagement Scale (UWES). The MLQ‐5X, consisting of 45 items divided into 12 sub‐scales, assessed leadership styles on a five‐point Likert scale, while the UWES, with 17 items rated on a seven‐point Likert scale, measured work engagement across three sub‐scales: vigour, dedication and absorption. Both instruments have demonstrated good reliability and validity in previous research (Aboshaiqah et al. 2016; Al‐Yami et al. 2018). Data collection occurred from November 2022 to January 2023 using both online (Qualtrics survey platform) and hardcopy questionnaires. Surveys were disseminated via email, WhatsApp and posters with QR codes and secure boxes were provided for hardcopy returns. A total of approximately 1000 nurses were employed across the four hospitals. Among them, 450 nurses volunteered to participate in the survey, with 278 meeting the inclusion criteria and completing the survey, resulting in a response rate of 61.78%.

#### 2.4.2 Linking Quantitative to Qualitative Phase

The results from the quantitative phase informed the development of the qualitative phase, providing a broad understanding of the relationships between leadership styles and work engagement levels. Deeper exploration was needed to understand these statistical correlations, underlying reasons and experiences.

#### 2.4.3 Qualitative Data Collection

The qualitative phase employed a qualitative descriptive design to explore registered nurses' perceptions of the impact of nurse manager leadership on work engagement. Thirteen one‐on‐one semi‐structured interviews were conducted via zoom between July and August 2023, lasting 25–60 min each. The interviews were conducted in Arabic for Arabic‐speaking participants and in English for non‐Arabic speakers. Data saturation was reached when no new information emerged.

### 2.5 Data Analysis

#### 2.5.1 Quantitative Analysis

Quantitative data were analysed using IBM SPSS Statistics (version 27). Descriptive statistics (means, standard deviations and frequencies) summarised demographic characteristics, leadership styles and engagement levels. Inferential tests, including independent *t*‐tests, ANOVA and Pearson correlation coefficients, were used to examine relationships between leadership styles and nurse work engagement.

Assumptions for parametric and regression analyses were examined and confirmed prior to hypothesis testing. Normality was assessed using skewness and kurtosis values, all of which fell within acceptable limits (±1.5). Homogeneity of variances was verified using Levene's test (*p* > 0.05). Multicollinearity was evaluated using Variance Inflation Factor (VIF) values, all below 3, indicating no multicollinearity concerns.

Regression assumptions were further assessed through SPSS diagnostic procedures. Inspection of residual plots confirmed linearity and homoscedasticity, and standardised residual distributions supported normality. Independence of errors was verified using the Durbin–Watson statistic, which fell within the acceptable range. These diagnostic checks indicated that all assumptions were sufficiently met for the use of parametric and regression analyses.

#### 2.5.2 Qualitative Analysis

Interview transcripts were analysed using thematic analysis. The process involved multiple readings, initial coding and the development of themes through constant comparison. Coding was done inductively to capture emerging patterns, and themes were refined collaboratively by the research team to ensure rigour and confirmability.

### 2.6 Mixed Methods Integration

Integration of quantitative and qualitative findings was conducted using the Pillar Integration Process (PIP) (Johnson et al. 2019). This structured approach enabled a systematic synthesis of data across methods and included the following steps:

1. Listing and sorting (Pillars 1 & 5): Quantitative codes were listed in Pillar 1, and qualitative excerpts were compiled in Pillar 5.
2. Creating categories (Pillars 2 & 4): Each dataset was thematically organised into categories that captured commonalities and differences
3. Data integration (Pillar 3): Central, cross‐cutting themes were generated by comparing and synthesising categories from both datasets
4. Narrative development: The integrated themes were used to construct an overarching narrative explaining how nurse manager leadership styles influence work engagement.

Themes were independently reviewed and agreed upon by multiple researchers to ensure analytical credibility. The PIP framework enhanced methodological transparency and supported the development of a cohesive explanation grounded in both numerical trends and lived experiences.

### 2.7 Ethics

Ethical approval was obtained from the relevant ethics committees. All participation in the study was voluntary. Principles of informed consent, confidentiality and no harm were maintained throughout the research. Participants had the right to withdraw from the study at any time.

## 3 Results

### 3.1 Demographic Characteristics of the Participants

This study included 278 participants, the majority of whom were female (227, 81.7%). Over half of the participants were non‐Saudi expatriates (167, 60.1%), primarily from the Philippines and India (35, 12.0%), with smaller numbers from Egypt, Jordan, Tunisia, Nigeria, Malaysia, Indonesia and Sudan. Most respondents held a Bachelor of Science in Nursing (BSN) degree (205, 73.7%), had less than six years of work experience (111, 39.9%), were aged between 30 and 39 years (176, 63.3%) and were employed in inpatient units.

#### 3.1.1 Quantitative Key Findings

Results showed significant positive correlations between transformational leadership and work engagement (*r* = 0.65, *p* < 0.01, 95% CI [0.58, 0.71]), indicating that nurses who perceived their managers as transformational reported higher levels of dedication, vigour and absorption. Transactional leadership also had a moderate positive correlation with work engagement (*r* = 0.56, *p* < 0.01, 95% CI [0.47, 0.64]), while passive‐avoidant leadership negatively correlated with work engagement (*r* = −0.12, *p* < 0.05, 95% CI [−0.23, −0.002]), suggesting that nurses working under passive or avoidant managers exhibited higher levels of disengagement.

A one‐way ANOVA was conducted to compare work engagement scores across leadership style categories. Results indicated statistically significant differences between the three leadership styles: transformational leadership *F*(2, 275) = 42.68, *p* < 0.001, *η* ^2^ = 0.237; transactional leadership *F*(2, 275) = 27.35, *p* < 0.001, *η* ^2^ = 0.166; and passive‐avoidant leadership *F*(2, 275) = 4.21, *p* = 0.016, *η* ^2^ = 0.030. These results demonstrate that nurses who perceived higher levels of transformational and transactional leadership reported significantly greater work engagement, whereas passive‐avoidant leadership was associated with lower engagement levels.

A multiple linear regression analysis was performed to determine whether leadership styles predicted work engagement after controlling for demographic variables (age, nationality and years of experience). The overall model was statistically significant, *F*(6, 271) = 40.25, *p* < 0.001, explaining 47% of the variance in work engagement (*R* ^2^ = 0.47, adjusted *R* ^2^ = 0.45, AIC = 712.84, BIC = 739.27). Transformational leadership was the strongest positive predictor (*β* = 0.52, *p* < 0.001), followed by transactional leadership (*β* = 0.28, *p* = 0.002). Passive‐avoidant leadership showed a weak negative association (*β* = −0.11, *p* = 0.041). Among demographic variables, nationality contributed significantly (*β* = 0.09, *p* = 0.034), indicating that Saudi nurses reported slightly higher engagement levels than non‐Saudi nurses. Age and years of experience were not significant predictors in the adjusted model.

Independent‐samples *t*‐tests were conducted to examine differences in work engagement scores between Saudi and non‐Saudi nurses. The results showed that Saudi nurses reported slightly higher mean engagement levels (M = 3.71, SD = 0.48) than non‐Saudi nurses (M = 3.58, SD = 0.52), *t*(276) = 2.18, *p* = 0.031, 95% CI [0.012, 0.253]. Although the difference was statistically significant, the effect size was small (Cohen's d = 0.25), indicating a modest practical difference between groups.

Notable differences were observed between Saudi and non‐Saudi nurses, with non‐Saudi nurses reporting higher transformational leadership perceptions (*M* = 3.45, SD = 0.68) compared to Saudi nurses (*M* = 3.26, SD = 0.72; *t*(276) = −2.14, *p* = 0.033, Cohen's *d* = 0.32). Similarly, transactional leadership was perceived more favourably by non‐Saudi nurses (*M* = 3.36, SD = 0.70) than by Saudi nurses (*M* = 3.21, SD = 0.71; *t*(276) = −1.73, *p* = 0.09, Cohen's *d* = 0.23). Passive‐avoidant leadership was rated significantly higher among Saudi nurses (*M* = 2.74, SD = 0.80) compared to non‐Saudi nurses (*M* = 2.42, SD = 0.94; *t*(276) = 3.07, *p* = 0.002, Cohen's *d* = 0.45). Additionally, work engagement levels were significantly higher among non‐Saudi nurses (*M* = 4.23, SD = 1.00) than Saudi nurses (*M* = 3.73, SD = 1.07; *t*(276) = −4.00, *p* < 0.001, Cohen's *d* = 0.58).

#### 3.1.2 Qualitative Initial Results

The qualitative data was categorised into three primary themes, each with six subthemes. These themes included the impact of fair and equitable, equitable and culturally competent nurse managers, effective communication styles among nurse managers and supportive and collaborative nurse managers.

##### 3.1.2.1 Final Synthesis

**FIGURE 2:**
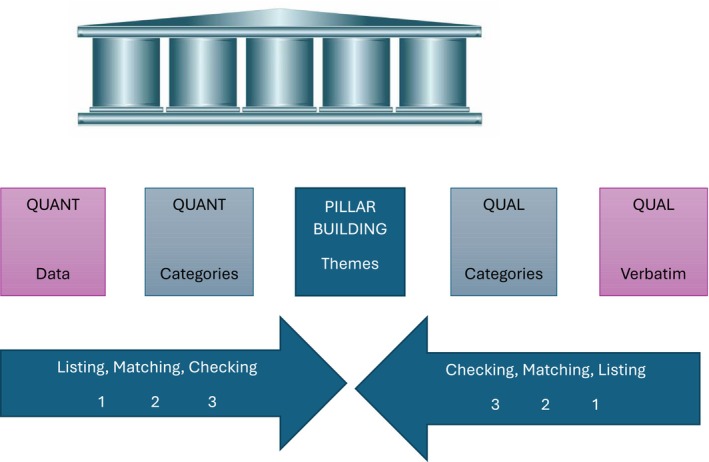
Representation of pillar integration process, example adapted from Johnson et al. (2019).

Figure 2 shows the representation of pillar integration process.

#### 3.1.3 Integrated Results

Using the Pillar Integration Process, four integrated themes were identified: relational Leadership and Engagement, Recognition and Reward‐Based Performance and Engagement, Negative Impact of Neglectful Leadership and Cultural Competence in Leadership.

Table 2 summarises the integrated findings using the Pillar Integration Process.

**TABLE 2:** Pillar integration process matrix.

Pillar 1	Pillar 2	Pillar 3	Pillar 4	Pillar 5
Quantitative data	Quantitative categories	Integrated themes	Qualitative categories	Qualitative verbatim
Transformational leadership Most common style (mean score 3.37, SD 0.70) Significant positive effect on nurse work engagement (*r* = 0.65, *p* < 0.01). Includes: Individualised consideration (24.80%), inspirational motivation (25.61%), intellectual stimulation (24.65%) and idealised influence (24.94%)	High prevalence Significant positive effect on engagement Components of transformational leadership Individualised consideration (24.80%): This involves leaders attending to each follower's needs, acting as mentors or coaches and providing personalised support	Relational leadership and engagement	Fair, equitable Nurse managers	*Our head nurse considered our vacation needs, allocating leave fairly, which made us truly appreciate our work—*Huda, CCU *The head of our department allocated tasks and cases based on individual strengths and workloads, enhancing my engagement and sense of belonging—*Abeer, ICU
	Intellectual stimulation (24.65%): Leaders encourage innovation and creativity, challenging followers to think critically and solve problems in new ways		Effective communication styles among nurse managers	*When a problem arises, the head nurse attentively listens to our issues and seeks solutions. This support enables us to excel in tasks, deepening our commitment to work—*Abeer, ICU *She often discusses every point with her staff. She would hold brief meetings with them to discuss issues and guide them in detail on tasks such as sending emails and receiving cases. There is communication. Despite the high workload in this unit, handling 8–9 cases per shift, the staff feel less stressed than those in other units with lower capacities—*Huda, CCU
	Idealised influence (24.94%): Leaders act as role models, gaining the trust and respect of their followers through their behaviour and ethics.		Supportive and collaborative nurse managers	*If a nurse comes to work stressed or faces a hard time, the head nurse asks her what is wrong and then supports her. This way, we all feel satisfied, more engaged, and dedicated to our work and each other.—*Julia, Surgical Unit *Our leader consistently supports us, even in conflicts with other professionals. Her unwavering support has made us deeply loyal; no one wants to leave her unit. We all recognise her value and reciprocate her support, ready to assist whenever she needs us—*Arwaa, Paediatric Unit
	Inspirational motivation (25.61%): Leaders communicate a compelling vision, inspiring and motivating followers to achieve more than they thought possible		Communicating recognition and appreciation	*When leaders appreciate your work, you can work properly and bear all the pressure at work because there is appreciation—*Mona, Oncology Unit *When a leader appreciates his employees, gives them their rights, and respects them, it makes a great difference… I come to work with love, feeling happy and enjoying working with her—*Sarah, ICU *It's different from one staff member to another. While some are motivated by self‐development, others appreciate small gestures like coffee or sweets. I seek a balance in my workload and value cooperation when I need a vacation; [then] I can do more work—*Jody, Paediatric Unit
Transactional leadership Second most common style (mean score 3.30, SD 0.71) Positive correlation with work engagement (*r* = 0.56, *p* < 0.01)	Moderate prevalence Moderate positive effect on engagement	Recognition and reward‐based performance	Reward‐based on performance	*When you know someone appreciates your work, you will find people working with dedication. They work from their heart, not just for a salary—*Nour, ER *If manager leaders are appreciative, we will be interested in getting involved in and engaged in our work—*Khalid, ICU *We will be more engaged if the manager shows appreciation. Good appreciation appraisals, increments, and incentives make physical and emotional stress manageable because we feel recognised and rewarded—*Abeer, ICU
Passive avoidant leadership Least common style (mean score 2.55, SD 0.90) Negative correlation with work engagement (*r* = −0.12, *p* < 0.05)	Low prevalence Negative effect on engagement	Negative impact of neglectful leadership	Lack of support	*We have a lot of compulsory leaves and days off … I can [theoretically] take 23 days off but they expired and the unit hasn't compensated us. Now they don't give us money for it; they give us extra time…—*Nour ER
Differences between Saudi and non‐Saudi nurses Non‐Saudi nurses report higher scores for transformational leadership (3.45 ± 0.68) compared to Saudi nurses (3.26 ± 0.72) Non‐Saudi nurses have higher scores for transactional leadership (3.36 ± 0.70) compared to Saudi nurses (3.21 ± 0.71) Saudi nurses report higher scores for passive avoidant leadership (2.74 ± 0.80) compared to non‐Saudi nurses (2.42 ± 0.94)	Higher transformational leadership scores for non‐Saudis Higher transactional leadership scores for non‐Saudis Higher passive avoidant leadership scores for Saudis	Cultural competence in leadership	Culturally competent nurse managers	*During Eid, managers expect you to stay instead of returning to your family—*Sarah, ICU *Non‐Saudi leaders don't care about national holidays … Saudi leaders give us our rights as Saudis.—*Arwaa, Paediatric Unit *She used to ask me to work every weekend … When I tried to talk to her, she didn't understand.—*Arwaa, Paediatric Unit *Non‐Saudi leader tends to be biased toward her group … When I, as a Saudi woman, had a request, she would tell me ‘No’—*Nour, ER

### 3.2 Theme 1: Relational Leadership and Engagement

The study found that the transformational leadership style significantly drives nurse work engagement, with a notable mean score of 3.37 (SD = 0.70). Pearson's correlation test revealed a strong positive relationship between transformational leadership and work engagement (*r* = 0.65, *p* < 0.01, 95% CI [0.58, 0.71]), indicating a large effect size. Nurses who experienced the four key elements of transformational leadership—idealised influence, inspirational motivation, intellectual stimulation and individualised consideration—reported significantly higher levels of engagement. This leadership style was the most common and had the strongest positive correlation with work engagement.

The qualitative data reinforced these findings, demonstrating that nurses felt more engaged when leaders communicated effectively, provided support and treated employees fairly. For instance, individualised consideration was evident in nurses' experiences of feeling valued when managers considered their personal needs. Participant H1 (CCU) shared:

Our head nurse considered our vacation needs, allocating leave fairly, which made us truly appreciate our work.

This illustrates how leaders who recognise individual needs and promote fairness contribute to increased nurse engagement, aligning with the quantitative finding that transformational leadership positively impacts engagement.

Similarly, intellectual stimulation encouraged nurses to think critically and engage more actively in problem‐solving. Participant A2 (ICU) described:

When a problem arises, the head nurse attentively listens to our issues and seeks solutions. This support enables us to excel in tasks, deepening our commitment to work.

This supports the statistical finding that transformational leadership fosters engagement by encouraging innovation and collaboration.

The role of inspirational motivation and idealised influence in building trust and engagement was also highlighted. Participant J3 (Surgical Unit) stated:

If a nurse comes to work stressed, the head nurse asks her what is wrong and then supports her. This way, we all feel satisfied, more engaged and dedicated to our work and each other.

These findings illustrate that transformational leadership—particularly when expressed through relational behaviours such as fairness and support—fosters engagement by promoting a sense of belonging, trust and motivation in the workplace.

### 3.3 Theme 2: Recognition and Reward‐Based Performance

The moderate positive correlation between transactional leadership and work engagement (*r* = 0.56, *p* < 0.01, 95% CI [0.47, 0.64]) suggests that clear expectations, performance‐based rewards and structured incentives contribute to nurse motivation. Transactional leadership is based on rewarding good performance and correcting poor performance, creating an environment where nurses understand what is expected of them and what they will receive in return.

The quantitative findings indicate that nurses who receive recognition, financial rewards or structured incentives report higher levels of engagement. This relationship is reflected in nurses' experiences. Participant K1 (ER) shared:

When you know someone appreciates your work, you work with dedication—not just for a salary.

This illustrates how managerial recognition reinforces motivation and work engagement, aligning with the statistical evidence that transactional leadership positively affects engagement.

Similarly, Participant M3 (ICU) emphasised the role of structured reward systems:

If manager leaders are appreciative, we will be more engaged in our work.

However, not all nurses are equally motivated by financial incentives or formal recognition. Some nurses value smaller, more personal rewards or work‐life balance as key engagement factors. Participant Jody (Paediatric Unit) shared:

It's different from one staff member to another. While some are motivated by self‐development, others appreciate small gestures like coffee or sweets. I seek a balance in my workload and value cooperation when I need a vacation; [then] I can do more work.

This statement suggests that transactional leadership is effective for some nurses, but engagement levels may vary depending on individual preferences for motivation. While some nurses are driven by financial rewards or formal recognition, others value flexibility, cooperation and small incentives as engagement drivers.

These findings confirm that transactional leadership enhances engagement through structured incentives and recognition, but its effectiveness depends on how well leaders accommodate different motivational needs. While some nurses thrive under a performance‐reward system, others seek flexibility, teamwork and emotional support, suggesting that a balanced leadership approach may be necessary.

### 3.4 Theme 3: Negative Impact of Neglectful Leadership

The negative correlation between passive‐avoidant leadership and nurse work engagement (*r* = −0.12, *p* < 0.05, 95% CI [−0.23, −0.002]) suggests that this leadership style weakens motivation, reduces commitment and contributes to workplace dissatisfaction. Passive‐avoidant leaders tend to avoid making decisions, fail to provide guidance and neglect staff concerns, leading to low engagement and increased stress among nurses.

Nurses reported feeling unsupported and undervalued. Participant N1 (ER) shared:

We have a lot of compulsory leaves and days off … but they expired without compensation. Now, they don't even pay for it.

This reflects managerial neglect, reinforcing the statistical evidence that passive leadership negatively affects engagement.

Additionally, poor leadership communication led to resistance and disengagement. Participant Nour (ER) described:

In the Paediatrics unit, a head nurse known for harsh orders and a lack of listening skills faced widespread resistance. Most nurses tended to ignore her orders, resulting in absences and a severe staff shortage.

These findings confirm that passive‐avoidant leadership significantly undermines engagement, leading to burnout, absenteeism and dissatisfaction.

Similarly, nurses reported frustration over excessive workloads and a lack of leadership support. Participant F3 (ICU) shared:

The workload is overwhelming, and no one listens to our concerns. We're just expected to manage, no matter how short‐staffed we are.

This supports the quantitative findings that passive leadership fails to address workplace challenges, leading to burnout and reduced job satisfaction.

Furthermore, the emotional toll of passive leadership was evident in nurses' experiences of feeling ignored and undervalued. Participant R5 (Surgical Unit) stated:

When you feel ignored, unappreciated, and overworked, it's hard to stay motivated. It affects our mental health and makes us think about leaving.

### 3.5 Theme 4: Cultural Competence in Leadership

The study found notable differences in engagement levels based on nationality and cultural background, with non‐Saudi nurses reporting higher engagement levels than Saudi nurses. Non‐Saudi nurses had higher scores for transformational leadership (*M* = 3.45 ± 0.68) and transactional leadership (*M* = 3.36 ± 0.70) compared to Saudi nurses (*M* = 3.26 ± 0.72 and *M* = 3.21 ± 0.71, respectively). Conversely, Saudi nurses reported higher scores for passive‐avoidant leadership (*M* = 2.74 ± 0.80) compared to non‐Saudis (*M* = 2.42 ± 0.94).

The quantitative findings suggest that cultural competence among nurse managers plays a significant role in engagement levels. Nurses who perceived their leaders as culturally aware and inclusive reported higher engagement, while those who experienced cultural insensitivity or favouritism reported lower engagement levels.

This relationship is echoed in nurses' experiences. Participant S1 (ICU) highlighted how cultural insensitivity in scheduling led to frustration:

During Eid, managers expect you to stay instead of returning to your family.

This statement reflects a lack of cultural awareness in leadership practices, reinforcing the quantitative finding that cultural competence influences engagement. Nurses who feel their personal and cultural needs are overlooked may experience lower job satisfaction and motivation.

Conversely, some nurses perceived Saudi leaders as more culturally understanding, which enhanced engagement. Participant A2 (Paediatric Unit) stated:

Non‐Saudi leaders don't care about national holidays … Saudi leaders ensure we get our rights as Saudis.

This finding suggests that leaders who acknowledge and respect cultural traditions contribute to a more inclusive and supportive work environment, fostering higher engagement among their culturally similar staff.

Additionally, some nurses reported the perception of favouritism and bias based on nationality, which influenced their engagement levels. Participant N3 (ER) shared:

Non‐Saudi leaders tend to be biased toward their group … When I, as a Saudi woman, had a request, she would tell me ‘No.’

This experience illustrates how perceived bias or exclusion based on cultural background can negatively impact engagement, reinforcing the statistical finding that Saudi nurses reported lower engagement levels when working under non‐Saudi leaders.

These findings confirm that cultural competence in leadership is a crucial factor influencing engagement in diverse healthcare settings. Nurses who feel respected, valued and included by their leaders are more engaged, while those who perceive cultural insensitivity, favouritism or lack of recognition are at risk of disengagement and job dissatisfaction. This highlights the importance of leadership training in cultural competence to ensure a more inclusive and supportive workplace for all nurses.

## 4 Discussion

This mixed‐method study aimed to provide a comprehensive understanding of how different leadership styles influence nurse engagement in Saudi Arabia's multicultural healthcare environment addressing a gap regarding the interplay between cultural context and leadership effectiveness. While leadership is broadly defined as the ability to influence others to achieve shared goals (Northouse 2021), its application varies across cultural and organisational contexts, shaping how it is perceived and practiced (Cardiff et al. 2023). Our study identified several key themes—relational Leadership and Engagement, Recognition and Reward‐Based Performance, the Negative Impact of Neglectful Leadership and cultural competence in Leadership—that offer valuable insights into how nurse managers can promote work engagement among nursing teams.

### 4.1 Leadership Styles and Work Engagement

The study identified transformational leadership as the most prevalent and impactful style, with a strong positive correlation to nurse engagement (*r* = 0.65, *p* < 0.01), suggesting that nurses who perceive their leaders as transformational report significantly higher levels of work engagement. Transformational leadership, characterised by inspirational motivation, individualised consideration, intellectual stimulation and idealised influence, was consistently associated with increased motivation, job satisfaction and professional commitment. These findings are supported by qualitative data, where nurses described feeling more engaged when leaders communicated effectively, demonstrated fairness and provided emotional support. Participants highlighted that being listened to, treated with respect and encouraged to contribute fostered a sense of value and connection to their work.

Our findings align with existing literature that emphasises the importance of leadership practices promoting relational connectedness, professional autonomy and healthy workplace cultures (Cardiff et al. 2023). Relational connectedness, a key component of person‐centred and transformational leadership, involves creating supportive relationships that foster feelings of respect, appreciation and belonging (Cardiff et al. 2023; van der Borg et al. 2017). Leaders who build trust and foster a supportive environment contribute to higher nurse satisfaction and engagement, resulting in tangible benefits such as improved retention rates, reduced turnover and better patient outcomes (Abdullatif Ibrahim et al. 2023).

Moreover, transformational leadership behaviours of nurse managers—such as inspiring, intellectually stimulating and individualised consideration—are associated with numerous positive outcomes for nurses, patients and healthcare organisations (Goens and Giannotti 2024; Iqbal et al. 2019; Rajabi et al. 2017). These outcomes include creating a supportive work environment, promoting fairness and equity, fostering a strong culture of patient safety and improving job satisfaction (Rajabi et al. 2017). Nurse managers who inspire and motivate their staff not only enhance engagement but also cultivate a workplace where nurses feel valued and supported, which directly improves patient care and organisational effectiveness (Boamah et al. 2018).

The preference for transformational leadership among nurse managers is well‐documented (Ferreira et al. 2022; Goens and Giannotti 2024; Perez‐Gonzalez et al. 2024), with evidence suggesting that relationship‐focused leaders significantly enhance nurses' intent to stay and overall job satisfaction (Lega et al. 2017; Perez‐Gonzalez et al. 2024). Our study was guided by Bass's transformational leadership model (Bass and Riggio 2006), which categorises leadership styles along a continuum from transformational to laissez‐faire. Transformational leaders encourage a higher level of awareness regarding shared goals and values, motivating nurses to prioritise organisational objectives over personal self‐interest (Ystaas et al. 2023).

While transformational leadership was the dominant style observed in our study, transactional leadership also showed a moderately positive effect on nurse engagement. Transactional leadership, which emphasises performance‐based recognition, provided structure and a sense of accomplishment for nurses. However, it did not address the emotional and psychological needs of nurses as effectively as transformational leadership, which offers empathy, intellectual stimulation and long‐term engagement strategies. Prior research similarly suggests that transactional leadership is useful for maintaining short‐term performance but lacks the long‐term engagement benefits of transformational leadership (Breevaart et al. 2014).

Consistent with Bass's theory that leaders can exhibit both transformational and transactional characteristics (Bass and Riggio 2006), our findings show that while transactional leadership addresses short‐term goals, transformational leadership fosters creativity, emotional well‐being and a sense of purpose. Combining both leadership styles—clear performance expectations from transactional leadership and emotional support from transformational leadership—emerges as the most effective approach (Breevaart et al. 2014). Nurse managers can adopt this blended leadership style by setting clear goals and providing recognition while also regularly engaging with their teams to offer emotional support and individualised feedback.

While transformational and relational leadership styles have been shown to enhance nurse engagement and improve patient care, our study also highlights the significant adverse effects associated with perceived ineffective leadership practices. Specifically, neglectful or toxic leadership behaviours—characterised by inadequate support, unfair treatment and poor communication—have been linked to decreased nurse morale, increased turnover intentions and compromised patient safety (Ahmed et al. 2024). A study by Labrague (2021) found that toxic leadership behaviours among nurse managers were strongly associated with increased nurse‐reported adverse events, including medication errors, patient falls and healthcare‐associated infections. These behaviours also correlated with a perceived decline in the overall quality of care provided (Farghaly Abdelaliem and Abou Zeid 2023; Labrague 2021). Furthermore, research indicates that such negative leadership styles contribute to emotional exhaustion and burnout among nurses, leading to higher absenteeism and a greater intention to leave the profession (Farghaly Abdelaliem and Abou Zeid 2023; Jiaqing et al. 2025). This not only affects the well‐being of nursing staff but also undermines the stability and effectiveness of healthcare organisations (Farghaly Abdelaliem and Abou Zeid 2023; Labrague 2021).

Findings from the integration paper emphasise the critical importance of relational and transformational leadership styles, prioritising empathy, communication and support. In contrast to neglectful leadership, effective leadership not only enhances nurse engagement but also safeguards the quality of patient care, underlining the necessity for nurse managers to foster positive, trust‐based relationships within their teams.

Although the negative correlation between passive‐avoidant leadership and work engagement was statistically significant (*r* = −0.12, *p* < 0.05), the effect size was small. This suggests that while the finding is statistically meaningful, its practical impact is limited. The result indicates that passive‐avoidant behaviours may contribute to disengagement, but other contextual and organisational factors likely play a more substantial role in influencing nurses' engagement levels.

#### 4.1.1 Cultural Competence in Leadership

Findings from this research found cultural competence emerged as a key factor in effective leadership, particularly in a multicultural environment such as Saudi Arabia. Transformational leadership's emphasis on individual consideration aligns with culturally competent leadership, which values the unique needs and backgrounds of team members. In this study, nurses highlighted the importance of culturally sensitive leadership in fostering an inclusive and engaging work environment, which in turn improved patient outcomes.

In culturally diverse healthcare settings, such as those in Saudi Arabia, leaders who respect and accommodate different cultural backgrounds are more likely to foster nurse engagement. According to Almutairi et al. (2015), the cultural values, social norms and national characteristics influence how people view their roles and interactions within a team. Healthcare organisations, particularly in multicultural settings, should integrate cultural awareness into leadership training and team‐building efforts (Almutairi et al. 2015; Dreachslin et al. 2012).

Although cultural factors were not the primary focus of this study, the observed variations in leadership preferences between Saudi and expatriate nurses suggest potential influences from cultural factors. The literature suggests that people's perceptions of leadership styles vary across cultures due to differences in authority structures, individualism vs. collectivism and gender roles (Hofstede 2011; House 2004). For example, Saudi Arabian culture is characterised by a high‐power distance, meaning that hierarchical structures are respected, and nurses from this background may prefer leaders who provide clear directives and maintain structure (Hofstede 2011); In contrast, expatriate nurses from more egalitarian cultures, such as those with lower power distance, may favour transformational leadership styles that emphasise participation and personal development (Almutairi et al. 2015; House 2004). Additionally, gender dynamics also play a role in leadership perception, especially in traditionally male‐dominated societies like Saudi Arabia (Al Mutair et al. 2023). As more women assume leadership roles in nursing, navigating these dynamics is crucial to ensuring that female leaders are respected and accepted across different cultural groups (Al Mutair et al. 2023; House et al. 2013). Understanding these cultural factors provides a valuable lens for interpreting this study's findings, especially regarding the effectiveness of transformational and transactional leadership styles in multicultural settings. Future studies could explore how cultural differences influence leadership preferences and work engagement among nurses in Saudi Arabia. Such research could help to further clarify the variations in engagement observed in this study and guide culturally tailored leadership training programmes in the healthcare sector.

## 5 Implications

### 5.1 Implications for Nursing Practice

This study highlights the critical role of leadership development in enhancing nurse engagement and retention. Healthcare organisations should implement structured leadership training programmes focused on developing transformational leadership competencies, including empathy, individualised support and inspirational motivation. Regular workshops and coaching sessions should be introduced to help nurse managers refine their leadership strategies and integrate relationship‐based leadership behaviours into daily practice.

Culturally competent leadership is essential, especially in multicultural environments like Saudi Arabia. Nurse leaders who understand and respect cultural differences enhance team cohesion and patient care. Training in cultural competence should be integrated into leadership development to address the needs of diverse teams effectively.

Addressing neglectful leadership is crucial for maintaining nurse morale, productivity and retention. Healthcare organisations should develop leadership accountability frameworks, ensuring that nurse managers receive training in effective communication, conflict resolution and staff recognition. 360‐degree feedback mechanisms, where nurses can provide anonymous feedback on leadership effectiveness, should be implemented to identify and address gaps in managerial support (Emam et al. 2024).

### 5.2 Implications for Nursing Education

To prepare future nurse leaders, nursing education curricula should integrate leadership training that blends transformational and transactional leadership approaches. This will equip nurses with the skills to set clear performance expectations (transactional leadership) while fostering trust and emotional engagement (transformational leadership). Leadership courses should include real‐world case studies, simulation‐based training and mentorship programmes to help students develop practical leadership skills before entering the workforce.

Cultural competence should also be embedded in nursing education, ensuring that future nurse leaders are equipped to manage diverse teams. Nursing programmes should include modules on cultural intelligence, communication styles and hierarchical structures across different healthcare systems.

These recommendations align closely with the goals of Saudi Vision 2030, which emphasises building a thriving healthcare sector driven by highly skilled, adaptable and culturally aware professionals. Specifically, Vision 2030 calls for investment in workforce development, leadership empowerment and health sector transformation to enhance the quality and efficiency of healthcare services. Equipping nurses with advanced leadership and cultural competence skills directly supports these objectives by fostering a capable nursing workforce that can lead within a modernised, patient‐centred health system.

In alignment with Saudi Vision 2030 health workforce transformation goals, the integrated findings provide direct guidance for leadership development in Saudi hospitals. Specifically, the relational, transformational and reward‐based behaviours identified in this study can be incorporated into nurse manager training workshops, leadership competency frameworks and MOH professional development programmes to strengthen engagement, support staff well‐being and improve workplace culture.

### 5.3 Implications for Nursing Research

Further research is needed to examine the long‐term impact of transformational and transactional leadership on nurse retention and patient care outcomes. Future studies should employ longitudinal designs to track how leadership training interventions influence nurse engagement over time. Additionally, cross‐cultural comparative studies can provide deeper insights into how leadership effectiveness varies across different healthcare systems.

Given the unique challenges of multicultural nursing teams, there is a need to develop leadership assessment tools that are tailored to diverse healthcare settings. Researchers should focus on designing context‐specific leadership evaluation frameworks that account for cultural differences in leadership perceptions and engagement preferences. These tools can help nursing administrators assess leadership effectiveness more accurately and refine training programmes accordingly.

### 5.4 Strengths and Limitations

This study provides novel insights into how leadership styles influence nurse engagement, using an explanatory sequential mixed‐methods design. Integrating quantitative and qualitative data allowed for both breadth and depth: surveys quantified leadership behaviours and engagement levels, while interviews explored nurses' lived experiences. Validated instruments enhanced reliability, and the Pillar Integration Process ensured robust synthesis of findings.

However, some limitations should be acknowledged. The use of convenience sampling and a relatively small sample size may limit generalisability. Participants with stronger views may have self‐selected, introducing bias. The cross‐sectional design restricts causal inference, and potential confounding variables cannot be fully ruled out. Additionally, the focus on government hospitals limits the applicability of findings to other healthcare sectors, such as private or military institutions. In addition, the use of a convenience sampling approach and the focus on nurses working in Saudi Arabia may limit the external validity and generalisability of the findings to other cultural and organisational contexts. Cultural norms, leadership expectations and workforce diversity may influence how leadership styles are perceived and how they impact engagement. Future studies should consider employing stratified or random sampling methods across diverse healthcare institutions and regions to enhance representativeness and allow broader comparison of leadership–engagement relationships.

## 6 Conclusion

To our knowledge, this is the first mixed methods study to address how nurse manager leadership positively influences nurse engagement in the workplace, organisation or profession. The integrated results provide a comprehensive understanding of nurse managers influencing nurse engagement through various leadership practices. Relational leadership and recognition are crucial for enhancing engagement, while neglectful leadership has severe negative impacts. Cultural competence is essential for effective leadership in diverse environments. These findings suggest that a multifaceted approach to leadership development, which includes fostering relational skills, recognising and rewarding performance, avoiding neglectful practices and enhancing cultural competence, is essential for improving nurse engagement and addressing the global nursing shortage. Future research should continue to explore these themes in different cultural and organisational contexts to develop tailored strategies for leadership development in healthcare.

## Funding

This study did not get specific funding. The lead researcher received a PhD candidate scholarship sponsored by Umm Al‐Qura University.

## Conflicts of Interest

The authors declare no conflicts of interest.

## Data Availability

The data that support the findings of this study are available on request from the corresponding author. The data are not publicly available due to privacy or ethical restrictions.
